# Channel Dysfunction as the Basis for Comorbidities in Multiple Sclerosis and Depression

**DOI:** 10.1002/ardp.70273

**Published:** 2026-06-09

**Authors:** Nicole Rychlik, Kay Jüngling, Sven G. Meuth, Thomas Budde, Marta Méndez‐Couz

**Affiliations:** ^1^ Department of Neurology University Hospital Münster Münster Germany; ^2^ Institute of Physiology I, University of Münster Münster Germany

## Abstract

Multiple sclerosis (MS) is a chronic inflammatory demyelinating disease of the central nervous system that is frequently accompanied by major depressive symptoms, which substantially worsen disease burden and quality of life. Increasing evidence suggests that this comorbidity is not solely a psychological reaction to neurological disability but may arise from shared neurobiological mechanisms. Ion channels play a central role in regulating neuronal excitability, synaptic transmission and immune cell function, and their dysregulation has emerged as a key feature of MS pathology. Demyelination and neuroinflammation induce profound alterations in the expression, distribution and function of several ion channel families, including hyperpolarization‐activated cyclic nucleotide‐gated (HCN) channels, voltage‐gated potassium channels such as K_v_7 and two‐pore domain potassium (K_2P_) channels. These changes contribute to neuronal hyperexcitability, altered thalamocortical network activity and immune cell activation. Importantly, several ion channel systems implicated in MS are also associated with affective regulation, including TRPA1, ASIC1a, *N*‐methyl‐d‐aspartate receptors and serotonin 5‐HT3 receptors. This review summarizes current evidence linking ion channel dysfunction to both MS pathology and depression and highlights emerging pharmacological strategies targeting these channels. Understanding ion channel‐mediated mechanisms may provide novel therapeutic opportunities to address both neuroinflammation and neuropsychiatric comorbidities in MS.

## Graphical abstract

Ion channel remodelling induced by neuroinflammation in multiple sclerosis alters neuronal excitability and immune signalling, creating shared molecular pathways that link multiple sclerosis pathology to depression and reveal novel pharmacological targets.

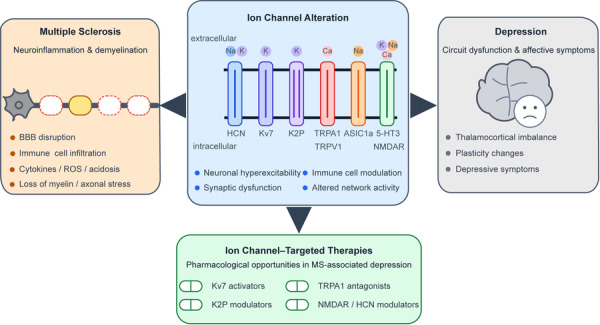

**Abbreviations**
4‐AP: 4‐aminopyridine
AQP4: aquaporin‐4
ASICs: acid‐sensing ion channels
BBB: blood brain barrier
cAMP: cyclic adenosine monophosphate
CNS: central nervous system
CSF: cerebrospinal fluid
DRF: diroximel fumarate
EAE: experimental autoimmune encephalomyelitis
EPSPs: excitatory postsynaptic potentials
HCN: hyperpolarization‐activated cyclic nucleotide‐gated cation
ICAM1: intracellular adhesion molecule 1
*I*_SO_
: standing outward current
K_2P_
: two‐pore domain potassium
KCNQ3: potassium voltage‐gated channel subfamily Q member 3
MBP: myelin basic protein
MOG: myelin oligodendrocyte glycoprotein
MS: multiple sclerosis
NMDARs: N‐methyl‐d‐aspartate receptors
NO: nitric oxide
PBMCs: peripheral blood mononuclear cells
PcTx1: psalmotoxin‐1
PIP_2_
: phosphatidylinositol 4,5‐bisphosphate
PLP: proteolipid protein
PPMS: primary progressive Multiple Sclerosis
RRMS: relapsing‐remitting Multiple Sclerosis
SPMS: secondary progressive Multiple Sclerosis
TASK: TWIK‑related acid‑sensitive K^+^ channel
TC: thalamocortical
THIK: two‐pore‐domain heak‐regulated ion K^+^ channel
TREK: TWIK‐related established K^+^ channel
TRIP8b: tetratricopeptide repeat Rab8b‐interacting protein
TRPA1: transient receptor potential ankyrin 1
TRPV1: transient receptor potential vanilloid 1
TWIK: tandem of whole cell inwardly K^+^ selective
VCAM1: vascular cell adhesion molecule 1
vMGN: ventral medial geniculate nucleus

## 1 Introduction

Multiple sclerosis (MS) and major depressive disorders are traditionally viewed as distinct clinical conditions. However, depression is one of the most common symptoms in patients with MS and significantly impacts the quality of life and the disease outcome. Current meta‐analytic data indicate that approximately 27% of people with MS show criteria for depression [1]. Depression appears more frequently in MS than in the general population and remains a leading cause of reduced quality of life and functional impairments [2] and can occur even at early stages of the disease [3, 4]. However, some disease‐modifying therapies for MS, particularly interferon‐based treatments, have been associated with depressive symptoms in some patients [5]. This high co‐occurrence points to shared neurobiological mechanisms that contribute to both disease processes.

MS is an inflammatory, chronic, demyelinating disease of the central nervous system (CNS), leading to functional deficits [6]. Dysfunction of ion channels that regulate the ion flow across the membrane is increasingly recognized as a key contributor to MS pathology [7]. Ion channels also play a crucial role in neuronal excitability and synaptic signalling in mood regulation, and growing evidence correlates ion channel dysfunction in the pathogenesis of depression [8]. Taken together, these observations point to an interplay between MS and depression at a level of ion channel biology. Understanding how specific ion channels are involved and contribute to these conditions could help to identify shared molecular pathways and targets for novel therapeutic strategies.

## 2 Channelopathy and Neuroinflammation: Understanding MS Beyond Immunity

During MS, demyelinating lesions emerge in both the brain and spinal cord, leading to degradation of myelin sheaths that surround axons, axonal injury and neuronal loss [9, 10]. While the cause of the disease is not fully investigated, the disease is considered a T‐cell‐driven disease. A dysregulation of the blood brain barrier (BBB) increases the infiltration of a variety of activated leucocytes, like macrophages, T‐cells and B‐cells into the CNS, where they target myelin‐derived self‐antigens, including myelin oligodendrocyte glycoprotein (MOG), proteolipid protein (PLP) and myelin basic protein (MBP), leading to further inflammation and demyelination, including loss of oligodendrocytes, reactive gliosis and neuro‐axonal degeneration [11, 12]. After invasion, they release pro‐inflammatory cytokines and chemokines that create an inflammatory environment, which recruits additional T‐cells, B‐cells, peripheral macrophages, and activate resident microglia and astrocytes [13].

MS affects approximately 2.8 million people globally, occurring most often in young adults and affecting twice as many women as men [6]. Although a minority of patients may experience early‐onset neurological symptoms during childhood or adolescence [14], prevalence differs markedly across countries, pointing to the role of environmental factors in shaping the clinical appearance of the disease. Smoking, vitamin D deficiency, obesity, exposure to ultraviolet B light [15], prior infection with viruses such as the Epstein‐Barr virus [16, 17, 18], and genetic factors [19] are recognized risk factors for this disease. Understanding these underlying risks helps to explore the different clinical courses and subtypes of MS, which reflect the diverse ways the disease progresses in affected individuals. Recently, ion‐channel disturbances have also been listed as a pathway involved in chronic neuronal and axonal injury [20].

MS is mainly classified into three main clinical subtypes based on symptom patterns and disease progression after the McDonald criteria [21, 22]. Relapsing–remitting MS (RRMS) is the most common subtype. It is characterized by episodic relapses followed by periods of remission and begins usually after an initial episode known as clinically isolated syndrome. During a relapse, increased permeability of the BBB enables the infiltration of autoreactive T‐cells into the CNS, leading to demyelination and neuronal injury. These pathological changes end up in a spectrum of symptoms, including optic neuritis, myelitis, sensory and motor dysfunction, fatigue, and cognitive impairments. Over time, about 80% of patients with RRMS transition to secondary progressive MS (SPMS), marked by continuous disability progression alongside relapses. In SPMS, ionic dysregulation disturbs mitochondrial activity and impairs neuronal excitability. The resulting cortical atrophy contributes to irreversible accumulation of cognitive and physical deficits. Approximately 10% of patients manifest primary progressive MS (PPMS), distinguished by gradual and continuous progression without distinct relapses.

Beyond the main clinical classification, MS is increasingly recognized as a channelopathy, in which dysregulated expression and redistribution of ion channels contribute to neurological symptoms [7]. Channelopathies represent a group of disorders characterized by abnormal ion channel function, leading to diverse clinical manifestations. They are commonly divided into four main categories. (1) Genetic channelopathies result from mutations that alter the structure or function of ion channels, causing either abnormal function or loss of function. (2) Autoimmune‐based channelopathies arise when elevated serum autoantibodies disturb the neuronal function or muscle ligand‐gated or voltage‐gated ion channels [23]. (3) Viral infections disrupt the regulation of cation channels such as Ca^2+,^ K^+^ and Na^+^, leading to imbalanced intracellular ion concentrations and altered cellular physiology [24]. (4) Transcriptional or translational channelopathies are linked to disturbed gene expression mechanisms that impair ion channel protein synthesis. The latter has been observed in cellular Purkinje neurons and may comprise their capacity for information encoding in both animal models and patients with MS [23]. Growing evidence suggests that MS can be classified into the fourth category, as dysregulation of ion channel gene expression contributes to disease pathophysiology [7].

## 3 Ion Channels as Mediators of Inflammation, Excitotoxicity and Repair in MS

Ion channels are fundamental to neuronal physiology by regulating the membrane potential, synaptic transmission, Ca^2+^ signalling and cell survival. In the CNS, different types of ion channels allow the flow of ions across the neuronal membrane. In MS, demyelination and axonal injury are associated with redistribution and altered expression of voltage‐gated Na^+^, K^+^, Ca^2+^ channels and hyperpolarization‐activated channels. These changes are now widely recognized as key contributors to MS‐related symptoms, such as fatigue, spasticity, pain and cognitive dysfunction. At the same time, they also highlight ion channels as promising therapeutic targets for MS [23].

To better understand how ion channels can influence neuronal behaviour in MS, it is helpful to consider different ion channel families.

## 4 HCN Channels

Hyperpolarization‐activated cyclic nucleotide‐gated cation (HCN) channels represent a key node linking demyelination‐induced network instability with affective circuit dysfunction. They are voltage‐gated cation channels responsible for conducting the *I*_h_ current and play a central role in regulating the membrane potential and oscillatory activity of neurons. They are known to conduct a depolarizing Na^+^ and K^+^ current, with the conductivity of the Na^+^ relying on the concentration of extracellular K^+^, which can change in demyelinating or inflammatory environments. HCN currents become inactive due to depolarization, thereby maintaining a steady resting membrane potential. They are activated by the binding of cyclic nucleotides, such as cyclic adenosine monophosphate (cAMP) [25]. This combination of gating allows HCN channels to regulate neuronal firing and network dynamics functions that are highly relevant to the disturbances seen in MS. Electrophysiological studies in the thalamocortical (TC) system observed a dysregulation of the *I*_h_ current in TC neurons after cuprizone induced demyelination in C57BL/6J mice [26, 27]. In addition, a combined genetic C3H/HeJ mouse model of human absence epilepsy, and pharmacological model, treated with cuprizone, investigated how acute global demyelination affects epileptic activity, myelin integrity and proinflammatory cytokines in the thalamus. Intrathalamic rhythmic bursting was significantly reduced, linked to changes in HCN channel function, specifically a decrease in the *I*_h_ current amplitude of TC neurons. Moreover, protein levels of HCN channels were reduced, along with a decrease in the phosphorylated form of tetratricopeptide repeat Rab8b‐interacting protein (TRIP8b), a regulatory protein that modulates HCN channel trafficking and function [28]. These findings are consistent with reported reduced burst activity in freely behaving mice following focal demyelination [29], which aligns with the reduced rhythmic bursting observed in brain slices after cuprizone treatment [28]. Combined with mathematical modelling, the data revealed a strong positive correlation between *I*_h_ availability and both the frequency and duration of intrathalamic bursting. Together, these results suggest that axonal demyelination leads to decreased burst activity through a reduction in *I*_h_ in thalamic neurons [29].

## 5 K_v_7 Channels

K_v_7 channels are key regulators of the neuronal M‐current that stabilize membrane excitability and their dysfunction has been implicated in the aberrant axonal signalling in MS. K_v_7.2 and K_v_7.3 are voltage‐gated potassium channels encoded by the KCNQ2 and KCNQ3 gene [30]. Together they form heterotetramers that mediate the neuronal M‐current, a slowly activating and non‐inactivating potassium current [31]. This current plays a critical role in regulating neuronal excitability by limiting action potential firing and controlling spike frequency adaptation. K_v_7.2 and K_v_7.3 channels are widely expressed in the central and peripheral nervous system at the axonal initial segments and nodes of Ranvier and are activated by phosphatidylinositol 4,5‐bisphosphate (PIP_2_) [32, 33]. Cuprizone‐induced demyelination leads to profound changes in the organization of K_v_7.3 ion channels at the nodes of Ranvier in neocortical layer V, which become either disintegrated or extended in the paranodal domains. Functionally, these structural changes had significant consequences, including lost saltatory conduction in demyelinated axons of layer V pyramidal neurons and parvalbumin‐positive interneurons, along with impaired presynaptic action potential firing and structural alterations at the axon initial segments and presynaptic terminals [34]. This kind of channel disorganization is not limited to the cuprizone model. Other studies in MS and experimental autoimmune encephalomyelitis (EAE) have demonstrated that both K_v_7.3 and K_v_7.2 channels become dysregulated within the TC system. Especially K_v_7.3 expression was significantly increased under acute and early inflammatory demyelinating conditions, suggesting a potentially compensatory response to altered excitability, since an EAE in mice is associated with neuronal hyperexcitability during the peak of the disease [35]. An altered excitability of neurons following demyelination or inflammation was already noted before. Cuprizone‐induced demyelination also alters neuronal firing patterns and synaptic transmission in the cortex. In this model, a reduction in the excitability of layer IV neurons was observed. These cells exhibited more hyperpolarized resting membrane potentials, long delays between stimulation and action potential firing, and reduced amplitudes of excitatory postsynaptic potentials (EPSPs) [36]. Another study used a lysolecithin model to induce focal lesions in the auditory cortex, leading to the demyelination of the injected area within a few days. Neurons in the ventral medial geniculate nucleus (vMGN), which play a key role in auditory processing, exhibit a dramatic reduction in burst firing. In addition, these neurons lost their frequency tuning as they lacked the ability to respond selectively to two distinct auditory tones. Similar results were observed when lysolecithin was injected directly into the vMGN. These findings suggest that demyelination disrupts thalamic firing patterns and simultaneously impairs sensory processing [29]. Also, differences in active membrane properties between K_v_7.3 (KCNQ3) knock‐out (ko) and wild‐type (wt) mice were found by measuring TC neurons' excitability. TC neurons recorded from MGN of K_v_7.3 ko mice fired a higher number of APs compared to wt mice. This finding clearly indicated that the K_v_7.3 subunits strongly influence active and passive membrane properties of TC neurons. Furthermore, in vivo electrophysiological recordings in vMGN in a longitudinal manner were performed in these mice. Two different tones were presented to the mice at 2.5 and 10 kHz, and changes in stimulus‐evoked neural activity were compared to baseline in the tonotopic region representing 10 kHz. Before EAE induction, wt and K_v_7.3 ko animals exhibited a similar low response to the non‐relevant stimulus (2.5 kHz). After EAE induction, an increased firing rate was observed in K_v_7.3 ko mice elicited at 10 kHz, indicating that EAE induction is associated with the emergence of hyperexcitability and promotes bursting discharge in vivo [37]. An altered K_v_7.2 expression is observed not only in neurons but also in peripheral blood mononuclear cells (PBMCs). When compared to healthy controls, MS patients showed a significant upregulation of K_v_7.2 channel protein expression in PBMCs. Notably, this increase appears to be sex‐specific. Female MS patients exhibit significantly higher protein levels compared to both healthy controls and male MS patients. Moreover, women with MS demonstrate a clear overexpression of K_v_7.2 relative to men with MS, suggesting a potential sex‐dependent regulation of K_v_7.2 expression in the disease context [38].

## 6 K_2P_ Channels

Members of the two‐pore domain potassium (K_2P_) channel family are also involved in MS pathogenesis. These channels are a distinct family of potassium channels that generate the so‐called background or leak K^+^ current and contribute to the standing outward current (*I*_SO_), which is essential for stabilizing the resting membrane potential and regulating cellular excitability in neurons [39]. They are encoded by the KCNK gene family and grouped into several subfamilies, including K_2P_1.1 (TWIK‐1), K_2P_6.1 (TWIK‐2), K_2P_3.1 (TASK‐1), K_2P_9.1 (TASK‐3), K_2P_15.1 (TASK‐5), K_2P_2.1 (TREK‐1), K_2P_10.1 (TREK‐2), K_2P_18.1 (TRESK), and K_2P_13.1 (THIK‐1), K_2P_12.1 (THIK‐2) channels. Depending on the subtype, they can be regulated by extracellular and intracellular pH, mechanical stretch, temperature, lipids, and inflammatory mediators. Through these properties, K_2P_ channels act as an important integrator of environmental and metabolic signals [40]. A connection between K_2P_ channels and MS has been demonstrated in a study showing increased mRNA expression of K_2P_3.1 (TASK‐1) and K_2P_9.1 (TASK‐3) channels in the optic nerve of EAE rats, whereas reduced expression was demonstrated in the thalamus and spinal cord. In addition, K_2P_9.1 (TASK‐3) mRNA was not only detected in neurons but also in immune cells within lesions of MS patients, suggesting a potential involvement of this channel in both neuroinflammation and neuronal processes in MS [41]. Following EAE induction, K_2P_3.1 (TASK‐1) ko mice exhibited significantly reduced clinical disease severity and markedly less axonal degeneration compared with wt controls. Furthermore, T‐cells in these mice showed impaired proliferation and cytokine production, indicating K_2P_3.1 (TASK‐1) as a potential molecular target for the therapy of demyelinating CNS disorders [42]. Similar results were found in an EAE induction in Lewis rats. Retreatment of MBP‐specific encephalitogenic T‐lymphocytes with K_2P_3.1 (TASK‐1) and K_2P_9.1 (TASK‐3) channel modulators led to an improvement in disease progression [43]. Also, genetic deletion of the K_2P_9.1 (TASK‐3) gene resulted in an ameliorated disease course in EAE mice [44]. Another K_2P_ channel, K_2P_2.1 (TREK‐1), appears to regulate immune cell trafficking across the BBB during chronic autoimmune neuroinflammation. In vitro experiments demonstrated that murine lymphocytes migrate more easily across endothelial monolayers derived from K_2P_2.1 (TREK‐1) ko mice compared to wt controls. Consistent with these findings, K_2P_2.1 (TREK‐1) ko mice developed a more severe disease phenotype in the EAE model, which is accompanied by increased numbers of CNS‐infiltrating T‐cells and elevated endothelial expression of the adhesion molecules ICAM‐1 and VCAM‐1 and actin network remodelling [45, 46]. Importantly, K_2P_2.1 (TREK‐1) expression is reduced in the microvascular endothelium within CNS lesions in MS patients [45]. Further investigation is needed, particularly given that BBB dysfunction is also implicated in other neurological and psychiatric disorders such as depression [47, 48]. To study the involvement of T‐cell and other K_2P_ channels like K_2P_5.1 (TASK‐2) in MS, its expression was studied in immune cells, PBMCs and cerebrospinal fluid (CSF) cells from MS patients. Human T‐lymphocytes were found to constitutively express K_2P_5.1(TASK‐2) with time‐dependent upregulation following T‐cell activation. Functional studies demonstrated that pharmacological inhibition with siRNA‐mediated silencing of K_2P_5.1 (TASK‐2) significantly impaired T‐cell effector functions, whereas overexpression enhanced T‐cell activity. Furthermore, electrophysiological recordings enabled a clear discrimination of K_2P_5.1 (TASK‐2) mediated currents from other K^+^ channels, confirming its important functional role in T cells. Importantly, the clinical relevance of these findings is underscored by altered K_2P_5.1 (TASK‐2) expression in MS patients. A significant upregulation of K_2P_5.1 (TASK‐2) was observed in CD4^+^ and CD8^+^ T‐cells from patients with RRMS during active phases. Elevated K_2P_5.1 (TASK‐2) levels were also detected on CD8^+^ T‐cells in patients with RRMS and SPMS, even during clinically stable phases. Furthermore, K_2P_5.1 (TASK‐2)‐positive T‐cells were identified within inflammatory lesions in MS brain tissue. Collectively, these findings highlight K_2P_5.1 (TASK‐2) as a functionally relevant regulator of T‐cell activity with clear pathophysiological implications in MS [49]. All of these studies show that ion channels emerged as critical regulators of immune cell function and CNS excitability in MS.

## 7 P2X7 Receptors

MS is characterized by inflammatory demyelination and progressive neurodegeneration in both white and grey matter. Within this context, activation of the ionotropic purinergic P2X7 receptors (P2X7R) on microglia and astrocytes contributes to the disease pathology by enhancing inflammatory responses, promoting lesion formation and driving damage to oligodendrocytes, ultimately leading to neuronal loss [50]. Post‐mortem studies of MS brain tissue have further demonstrated P2X7R expression on reactive astrocytes within lesions, while in vitro stimulation of astrocytes with P2X7R has been shown to increase nitric oxide synthase activity, suggesting a role in amplifying local neurotoxicity [51]. A distinctive feature of the P2X7R is its dual gating behaviour. Whereas low levels of ATP open a cation channel permeable to Na^+^, K^+^ and Ca^2+^, high ATP exposure promotes the formation of a larger, non‐selective high‐conductance pore. Prolonged activation of P2X7R induces the subsequent release of pro‐inflammatory cytokines such as IL‐1β, contributing to demyelination and axonal damage [51]. Evidence from the EAE model supports a pathogenic role for P2X7R, as its pharmacological inhibition attenuates disease severity and reduces immune cell infiltration [52]. In line with this, P2X7R has been found to accumulate with macrophages within EAE lesions [53], and increased P2X7R expression has been reported along axonal tracts in MS patients, where ATP‐mediated activation can induce oligodendrocyte death. Interestingly, P2X7R ko mice show increased susceptibility to EAE and enhanced CNS inflammation, indicating that P2X7R signalling may also play a context‐dependent and potentially regulatory role in neuroinflammatory diseases [54].

## 8 TRPV1 Channels

Transient Receptor Potential Vanilloid 1 (TRPV1) is a non‐selective cation channel with high permeability to Ca^2+^. It is expressed in small‐diameter sensory neurons and also in the central descending pathways that regulate nociception [55, 56]. TRPV1 channels have emerged as critical modulators of central inflammation in the pathogenesis of MS [57]. While MS is primarily characterized by demyelination and axonal loss, recent evidence suggests that the activation of TRPV1 receptors can facilitate myelin repair by regulating microglial function, offering a potential therapeutic target for remyelination [58]. Furthermore, Cannabidiol and other cannabinoids acting as an agonist/desensitizer interact with TRPV1 to modulate nociceptive signalling [59], and could potentially be used to manage MS‐related pain [60].

## 9 Targeting Ion Channel Dysfunction in MS

From a therapeutic perspective, drugs targeting ion channel function have shown promising results in slowing disease progression and reducing symptom severity. Both ion channel blockers and activators have demonstrated clinical potential, highlighting the translational potential of this approach. Different animal models of MS, like the EAE model, the cuprizone model and focal demyelination models, have been used to investigate the neuroprotective and immunomodulatory effects of these compounds. Retigabine, a K_v_7 channel activator, has been shown to restore TC neuron functionality in an EAE mouse model. They showed that Retigabine activated K_v_7 channels in control mice and in mice following EAE induction, leading to a reduced firing rate of neurons. Furthermore, Retigabine treatment following EAE induction improved the cognitive and neurological symptoms of the mice and prevented neurodegeneration in EAE [35]. The effect of Retigabine was also investigated in a K_v_7.3 ko model following induction of EAE, consistently showing reduced neuronal hyperexcitability even in K_v_7.3 ko mice through increasing the M‐current. Beyond its cellular electrophysiological effects, Retigabine also restored impaired auditory network functionality that emerged following EAE induction [37]. Furthermore, potassium channel blockage has also been demonstrated to have a therapeutic value. A number of reviews [61, 62, 63, 64] have already addressed the effect of 4‐aminopyridine (4‐AP), a voltage‐dependent potassium channel blocker in MS. 4‐AP enhances the conduction in demyelinating axons by reducing potassium efflux and improving action potential firing and synaptic transmission. Beyond voltage‐gated K_v_ channels, K_2P_ channels emerge as well as potential targets. The selective K_2P_3.1 (TASK‐1) channel inhibitor A293 showed a reduction of T‐cell function, such as cytokine production and proliferation. Importantly, this inhibitory effect was absent in CD4^+^ T‐cells derived from K_2P_3.1 (TASK‐1) ko mice but not in T‐cells of K_2P_9.1 (TASK‐3) ko mice. These results show that the effects were mediated by K_2P_3.1 (TASK‐1) rather than K_2P_9.1 (TASK‐3) channels. A293 reduced outward potassium currents in wt T‐cells but had no effect in K_2P_3.1 (TASK‐1)‐deficient cells, confirming K_2P_3.1 (TASK‐1) as the main target. In EAE mice, A293 reduced disease severity in wt mice, had no effect in K_2P_3.1 (TASK‐1) ko mice and remained partially effective in K_2P_9.1 (TASK‐3) mice [44]. Also, HCN channels represent a potential target. Different compounds were already tested and show promising results. EC18, for example, is a HCN4 channel blocker and significantly reduces the *I*_h_ current in the thalamus [65]. EC18 may reduce the observed increase of the *I*_h_ current in TC neurons following cuprizone‐induced demyelination in C57BL/6J mice [26, 27] and could serve as a potential treatment. With Diroximel fumarate (DRF), this effect has already been proven. DRF decreases the *I*_h_ current density during active remyelination [27]. Also, the sphingosine‐1 phosphate modulator Siponimod decreases the *I*_h_ current density in TC neurons [27] and furthermore improves cortical network functionality in an EAE mouse model [66]. These findings demonstrated that ion channel targeting in MS target both direct modulation of neuronal excitability and indirect effects through immune regulation and represent a promising neuroprotective strategy in MS.

## 10 Ion Channel Targets Linking MS and Depression

Several ion channel families have emerged as potential molecular links between MS pathology and depressive symptoms (Table 1). Among these, transient receptor potential ankyrin 1 (TRPA1) has been implicated in neuroinflammatory signalling and affective behaviour. In a progressive MS mouse model, TRPA1 activation was associated with the development of anxiety‐ and depression‐like behaviours, suggesting that inflammatory activation of this channel may contribute to mood disturbances during disease progression [78]. Beyond TRPA1‐mediated inflammatory signalling, changes in the extracellular environment during neuroinflammation can also activate other ion channel systems. Acid‐sensing ion channels (ASICs), particularly ASIC1a, represent another class of proton‐gated cation channels that respond to extracellular acidosis occurring during inflammatory processes. ASIC1a activation has been linked to neurodegeneration, pain processing and depression‐related behaviours, highlighting this channel as a potential therapeutic target in inflammatory CNS disorders, including MS [68, 69, 70].

**Table 1:** Ion channel targets potentially bridging MS and depression: pharmacological and drug discovery perspective.

Target	Channel class	MS‐relevant mechanism	Depression‐relevant mechanism	Pharmacological modulators/chemical tools	Reference
TRPA1	TRP non‐selective cation channel (Ca^2+^‐permeable)	Redox‐sensitive activation in inflammatory CNS (PMS‐EAE)	Associated with depression‐/anxiety‐like phenotype in MS model	A‐967,079 (antagonist)	[1]
TRPV1	TRP non‐selective cation channel (Ca^2+^‐permeable)	Regulates microglial activation, cytokine release and remyelination in experimental models	Modulates synaptic plasticity and neuronal excitability (preclinical evidence)	Capsaicin (agonist), capsazepine (antagonist)	[57, 58]
K_2P_2.1 (TREK‐1)	Two‐pore domain K^+^ (K_2P_) channel	Downregulation at inflamed BBB; regulates leucocyte trafficking	Controls neuronal excitability linked to mood regulation	Experimental K_2P_2.1 (TREK‐1) activators/inhibitors	[67]
ASIC1a	Proton‐gated Na^+^/cation channel	Implicated in inflammatory neurodegeneration	Linked to anxiety/depression‐like behaviour	Psalmotoxin‐1 (PcTx1); aromatic diamidines	[68, 69, 70]
NMDAR (GluN2C/2D)	Ionotropic glutamate receptor	Immune modulation; synaptic plasticity in CNS disorders	Established antidepressant target (glutamatergic modulation)	GluN2C/2D‐selective NAMs; ketamine; memantine	[71, 72]
5‐HT3 receptor	Ligand‐gated serotonin cation channel	Immunomodulatory signalling component in neuroinflammation	Modulates serotonergic neurotransmission in depression	5‐HT3 antagonists (ondansetron class)	[73]
HCN	Hyperpolarization‐activated cyclic nucleotide‐gated channel	Upregulation of *I*_h_ current	Reduced HCN function produces antidepressant‐like behaviour; disrupting TRIP8b‐HCN interaction reduces channel trafficking	ZD7288, TRIP8b‐HCN interaction inhibitors, Cilobradine	[74, 75]
P2X7R	Ionotropic purinergic receptor	Pro‐inflammatory cytokine release, oligodendrocyte death, axonal damage	Increase of oxidative stress and pro‐inflammatory cytokines	JNJ‐54175446 JMS06‐39	[52, 76, 77]

Abbreviations: BBB, blood brain barrier; CNS, central nervous system; EAE, experimental autoimmune encephalomyelitis; MS, multiple sclerosis.

In addition to TRPA1, other transient receptor potential channels, such as TRPV1 have been implicated in neuroinflammatory processes relevant to MS. Experimental studies demonstrate that TRPV1 modulates central inflammatory responses by regulating microglial activation and cytokine release, thereby influencing the extent of neuroinflammation in the CNS [58]. Furthermore, TRPV1 activation has been shown to facilitate remyelination following demyelinating injury through the modulation of microglial function, indicating a role in repair processes associated with MS pathology [57]. Beyond immune regulation, TRPV1 signalling also affects synaptic plasticity and neuronal excitability, suggesting a potential contribution to circuit‐level alterations that are relevant for affective symptoms. Taken together, these findings support a modulatory role for TRPV1 in neuroinflammatory and repair‐related mechanisms relevant to MS pathology. However, current evidence is largely restricted to experimental models, and direct clinical studies demonstrating a contribution of TRPV1 to depression in MS or therapeutic benefit from TRPV1‐targeted interventions are currently lacking.

Besides inflammation‐driven ion channel activation, altered neurotransmitter signalling further contributes to MS pathology and mood regulation. Glutamatergic signalling plays a central role in both MS‐related neurodegeneration and mood regulation. *N*‐methyl‐d‐aspartate receptors (NMDARs) regulate synaptic plasticity and excitatory neurotransmission, and pharmacological modulation of these receptors has demonstrated antidepressant effects. In particular, subunit‐selective modulation of GluN2C/2D‐containing NMDARs is therefore being explored as a potential therapeutic strategy for neurological and psychiatric disorders [71]. Furthermore, NMDAR signalling can influence immune cell function, as non‐competitive NMDAR antagonists have been shown to alter B‐cell activation and cytokine production [72].

Two‐pore domain potassium channels also contribute to this interface between neuroinflammation and neuronal excitability. The K_2P_ channel K_2P_2.1 (TREK‐1) regulates endothelial function and immune cell trafficking across the BBB, and reduced K_2P_2.1 (TREK‐1) expression in brain endothelial cells has been associated with enhanced leucocyte infiltration during neuroinflammatory conditions [67]. Genetic knock‐out or specific block of K_2P_2.1 (TREK‐1) with spadin results in an anti‐depressive phenotype [47, 79]. Finally, ligand‐gated serotonin 5‐HT3 receptors represent an additional ion channel system linking neuroinflammation and mood regulation. Pharmacological antagonism of these receptors has demonstrated neuroprotective and antidepressant effects, suggesting that serotonergic ion channels may contribute to the complex interplay between MS pathology and depression [73].

In addition to preclinical findings, emerging clinical evidence further supports a link between ion channel‐dependent network dysfunction and depressive symptoms in MS. Neurophysiological studies using electroencephalography or magnetoencephalography have consistently demonstrated alterations in brain oscillatory activity in MS patients, including reduced alpha power and changes in theta rhythms, which are associated with cognitive impairment and depressive symptoms [80, 81]. These oscillatory disturbances reflect altered neuronal excitability and synaptic integration, processes that are critically regulated by ion channels such as HCN channels, which contribute to rhythmic activity and pacemaker currents in TC circuits. Additionally, it is important to note that K_2P_2.1 (TREK‐1) is critical in regulating neuronal activity within the beta frequency band and that an increased power in this range is found under resting state conditions in MS [82] and depressive [83, 84] patients.

Complementary evidence from functional magnet resonance imaging studies indicates disrupted connectivity within fronto‐limbic and hippocampal networks in MS patients with depression, suggesting that large‐scale network instability underlies affective symptoms [85]. These network alterations are thought to reflect underlying changes in neuronal excitability and synaptic integration, processes that are disrupted in MS and associated with depressive symptomatology. Together, these findings suggest that ion channel‐dependent regulation of neuronal oscillations and network dynamics may represent a translational bridge between molecular mechanisms and clinical manifestations of depression in MS. Several of the chemical modulators summarized in Table 1 further support the role of ion channels as pharmacological interfaces between MS pathology and depression. Compounds targeting K_v_7, K_2P_ and related channel systems directly modulate neuronal activity and membrane stability. K_v_7 channel activation counteracts hyperexcitability associated with demyelination, while modulation of potassium channels by inflammatory mediators such as nitric oxide (NO) highlights their role in disease‐related neuronal dysregulation [86].

K_2P_2.1 (TREK‐1) also link immune and neuronal processes by regulating both neuronal responsiveness and BBB function [67]. Their involvement in depression‐related phenotypes suggests that K_2P_ channel modulators may exert both neuroprotective and antidepressant‐relevant effects. In addition, channel‐targeting compounds such as NMDAR modulators and 5‐HT3 receptor antagonists, have demonstrated antidepressant efficacy, supporting the idea that restoration of ion channel–dependent activity contributes to therapeutic effects [71, 72, 73].

Supporting evidence also suggests a link between ion channel dysfunction and the occurrence or comorbidity of depression in the context of MS. In an EAE model, voluntary activity, such as wheel running, delays disease onset and reduces pain hypersensitivity, indicating that activity‐dependent neuroplastic mechanisms modulate inflammatory disease progression [87]. Although ion channels were not directly interrogated, altered neuronal excitability likely contributes to early affective symptoms. Olechowski et al. [88] reported tactile allodynia and object recognition deficits in EAE mice, accompanied by spinal cytokine elevation and altered glutamate transporter 2 expression. These findings support glutamatergic imbalance as a component of MS‐associated behavioural dysfunction.

Beyond inflammatory and neurotransmitter‐related mechanisms, structural alterations within neurons may further influence ion channel function and excitability. Corticohippocampal dysfunction has been described in the OBiden mouse model of primary oligodendrogliopathy [89], suggesting that demyelination‐associated circuit disruption may involve compensatory redistribution of voltage‐gated sodium and potassium channels.

In addition to structural remodelling of neuronal circuits, changes in the extracellular environment can also affect ion channel localization and signalling. Extracellular matrix remodelling further contributes to excitability changes. Ulbrich et al. [90] describe dysregulation of perivascular and perineuronal extracellular matrix components in neurological and psychiatric diseases, including MS, potentially influencing ion channel localization and synaptic stability. Inflammatory mediators present in MS lesions may further modulate ion channel activity directly. NO signalling, for example, directly modulates ion channel function, particularly voltage‐gated potassium channels [86] and HCN channels [91]. Given increased inflammatory NO production in MS lesions, NO‐dependent channel modulation may contribute to mood circuit dysregulation. Although aquaporin‐4 (AQP4) is a water channel rather than a classical ion channel, it represents a key component of channelopathies in neuroinflammatory diseases such as neuromyelitis optica [92]. AQP4 is highly expressed in astrocyte endfeet surrounding blood vessels [93, 94] and plays a central role in water homoeostasis, BBB integrity and neuroimmune signalling [95]. While direct evidence linking AQP4 to depression in MS remains limited, its role in regulating the extracellular environment and astrocyte–neuron interactions indicates that it may indirectly influence neuronal excitability and network function. In AQP4 antibody‐associated disorders, disruption of astrocyte function leads to pronounced neuroinflammation and altered CNS homeostasis, highlighting the importance of astrocytic channel systems in inflammatory disease processes [92].

Importantly, clinical studies have linked AQP4 pathology with neurobehavioural symptoms. For example, patients with AQP4 antibody‐associated disease exhibit significant fatigue burden, suggesting that astrocyte dysfunction contributes to the manifestation of CNS inflammation [96]. Together, these findings suggest that, beyond classical ion channels, alterations in astrocytic membrane channels such as AQP4 may contribute to the interaction between neuroinflammation and neuropsychiatric symptoms.

Also, HCN channels emerge as potential therapeutic targets in both diseases. In depression, disrupting the interaction between HCN channels and the protein TRIP8b may selectively reduce HCN channel function in the brain [74]. Structural studies show that TRIP8b regulates HCN trafficking to distal dendrites of hippocampal CA1 pyramidal neurons, and loss of TRIP8b‐HCN binding impairs channel function [97]. Inhibiting HCN channels with Cilobradine and Cilobradine analogues represents a good basis for future antidepressant drug discovery [75]. Furthermore, the tetratricopeptide repeated domains of TRIP8b form a specific binding pocket, making them promising targets for small‐molecule inhibitors, while gene expression therapy has also been proposed to reduce hippocampal HCN expression [98]. Consistent with this, reduced hippocampal HCN activity has been associated with antidepressant‐like behaviour in animal models [99]. In MS, modulation of HCN channels may help stabilize neuronal excitability and improve conduction in demyelinated axons [26, 27], suggesting therapeutic relevance in neuroinflammatory diseases. Finally, safinamide illustrates the feasibility of multi‐target pharmacology, combining monoamine oxidase‐B inhibition with voltage‐sensitive sodium and potassium channel blockade [100], supporting a multi‐modal approach to disorders characterized by neuroinflammation and excitability imbalance.

Also, the P2X7R is emerging as an important mechanistic link between MS and depression. Whereas in MS these channels drive cytokine release and downstream inflammatory signalling [51], in depression P2X7R activation has additionally been associated with oxidative stress, mitochondrial dysfunction and disrupted synaptic plasticity, all of which may promote depressive pathology and stress sensitivity [101, 102, 103, 104]. Accordingly, pharmacological inhibition of P2X7R has emerged as a promising strategy for modulating neuroimmune mechanisms implicated in depressive disorders. JNJ‐54175446, a selective purine P2X7R antagonist, has been shown to attenuate microglial release of the pro‐inflammatory cytokines IL‐1β and IL‐18, thereby reducing downstream neuroinflammatory signalling. By limiting excessive microglial activation, P2X7R inhibition may counteract immune related dysregulation of mood and alleviate processes linked to stress vulnerability and depressive symptoms in situations where mood regulation is disturbed [76]. It can therefore be concluded that the development of P2X7R antagonists [77] provides new therapeutic opportunities for treating depression [76] and MS [52].

## 11 Conclusion and Perspectives

In summary, growing evidence suggests that ion channels play an important role at the intersection of MS pathology and depression. Ion channels regulate fundamental processes such as neuronal excitability, synaptic transmission and immune signalling, all of which are affected during neuroinflammatory conditions such as MS. A variety of ion channel and channel‐associated systems, including transient receptor potential channels, potassium channels, pacemaker channels, ligand‐gated channels and astrocytic channel regulators, have been linked to mechanisms that may contribute not only to MS‐related neurodegeneration but also to changes in mood and behaviour. These overlapping mechanisms may partly explain the high prevalence of depressive symptoms observed in patients with MS. Importantly, ion channels represent promising targets for new therapeutic approaches. Modulating ion channel activity has already shown beneficial effects in experimental models, including reduced inflammation, improved neuronal stability and antidepressant‐like outcomes. Since ion channels influence both immune processes and neuronal signalling, targeting these systems may offer the advantage of simultaneously addressing neurological pathology and neuropsychiatric comorbidities. Despite growing evidence linking ion channel dysfunction to both MS pathology and depression, several limitations should be considered when interpreting the current literature. Much of the available evidence is derived from experimental models, particularly EAE and other preclinical systems, which may not fully capture the complexity of human disease. In addition, the contribution of specific ion channel subtypes may vary across cell types, brain regions and disease stages, making it difficult to establish causal relationships. Finally, direct clinical studies examining the role of ion channel modulation in MS‐associated depression remain limited, highlighting the need for translational research to validate these mechanisms in patients. Nevertheless, several important questions remain. The specific role of individual ion channels across different cell populations and brain regions during MS progression is still incompletely understood, and their contribution to the development of depression in this context requires further investigation. Further studies, integrating neuroimmunology, electrophysiology and behavioural neuroscience will be essential to clarify these mechanisms. A deeper understanding of ion channel function in MS and depression may ultimately facilitate the development of therapeutic strategies that improve both neurological outcomes and quality of life for patients.

## Conflicts of Interest

The authors declare no conflicts of interest.

## Data Availability

Data sharing is not applicable to this article as no datasets were generated or analysed during the current study.
